# Factors Associated With Perceived Trust of False Abortion Websites: Cross-sectional Online Survey

**DOI:** 10.2196/25323

**Published:** 2021-04-19

**Authors:** Sarina Rebecca Chaiken, Lisa Han, Blair G Darney, Leo Han

**Affiliations:** 1 Oregon Health & Science University Portland, OR United States; 2 OHSU-PSU School of Public Health Portland, OR United States; 3 Arizona State University Tempe, AZ United States; 4 Center for Population Health Research National Institute of Public Health Cuernavaca Mexico

**Keywords:** abortion, website trust, internet use, reproductive health, misinformation

## Abstract

**Background:**

Most patients use the internet to search for health information. While there is a vast repository of searchable information online, much of the content is unregulated and therefore potentially incorrect, conflicting, or confusing. Abortion information online is particularly prone to being inaccurate as antichoice websites publish purposefully misleading information in formats that appear as neutral resources. To understand how antichoice websites appear neutral, we need to understand the specific website features of antichoice websites that impart an impression of trustworthiness.

**Objective:**

We sought to identify the characteristics of false or misleading abortion websites that make these websites appear trustworthy to the public.

**Methods:**

We conducted a cross-sectional study using Amazon’s Mechanical Turk platform. We used validated questionnaires to ask participants to rate 11 antichoice websites and one neutral website identified by experts, focusing on website content, creators, and design. We collected sociodemographic data and participant views on abortion. We used a composite measure of “mean overall trust” as our primary outcome. Using correlation matrices, we determined which website characteristics were most associated with mean overall trust. Finally, we used linear regression to identify participant characteristics associated with overall trust.

**Results:**

Our analytic sample included 498 participants aged from 22 to 70 years, and 50.1% (247/493) identified as female. Across 11 antichoice websites, creator confidence (“I believe that the creators of this website are honest and trustworthy”) had the highest correlation coefficient (strongest relationship) with mean overall trust (coefficient=0.70). Professional appearance (coefficient=0.59), look and feel (coefficient=0.59), perception that the information is created by experts (coefficient=0.59), association with a trustworthy organization (coefficient=0.58), valued features and functionalities (coefficient=0.54), and interactive capabilities (coefficient=0.52) all demonstrated strong relationships with mean overall trust. At the individual level, prochoice leaning was associated with higher overall trust of the neutral website (*B*=−0.43, 95% CI −0.87 to 0.01) and lower mean overall trust of the antichoice websites (*B*=0.52, 95% CI 0.05 to 0.99).

**Conclusions:**

The mean overall trust of antichoice websites is most associated with design characteristics and perceived trustworthiness of website creators. Those who believe that access to abortion should be limited are more likely to have higher mean overall trust for antichoice websites.

## Introduction

Most patients use the internet to search for health information [1-3]. While there is a vast repository of searchable information online, much of the content is unregulated and therefore has potential to be incorrect, conflicting, or confusing. As such, the accuracy of online health information remains variable [4]. Despite warnings that patients should remain cautious when accessing health-related websites, patients often view inaccurate health information encountered online as trustworthy [5,6].

Abortion is one of the most common medical services in the world, with approximately 30 million safe abortions accessed annually [7]. Although the abortion rate in the United States (abortions per 1000 females aged 15 to 49 years) has declined over the last decade, internet searches for abortion-related information have steadily increased [8,9]. As the demand for online abortion information rises, many top search results contain incorrect and misleading information [10]. Many misleading websites are created by crisis pregnancy centers (CPCs) and other antichoice organizations, which seek to dissuade patients from accessing abortion and other reproductive health care services [11]. These websites provide deliberately incorrect information about reproductive health, including abortion, often by overstating the risks of abortion and contraceptive options [12,13]. In addition, these antichoice organization websites often appear neutral in order to intercept patients who are potentially seeking an abortion [14].

Previous research identified website design and layout, interactive features, tone or partiality of content, owner’s authority, and the website’s relationship with an organization or sponsor as key elements impacting the perceived trustworthiness of a website [15-25]. Abortion websites may utilize these characteristics to make the misinformation they seek to perpetuate seem trustworthy. It is therefore important to understand what features of abortion websites impact trustworthiness. In this study, we sought to identify the characteristics of false or misleading abortion websites that make these websites appear trustworthy to the public.

## Methods

### Recruitment

We conducted a cross-sectional study using an anonymous online survey. We recruited respondents between March 14, 2019, and April 8, 2019, via Amazon’s Mechanical Turk (MTurk) platform. MTurk is an online crowdsourcing tool that recruits anonymous users to participate in a variety of computer-based tasks, including assessment of web content [26]. Participants were eligible to respond if they were over 18 years of age, English speaking, and given the “Masters Qualification” by MTurk. Amazon designates participants as “Masters” if they demonstrate reliability in completing a large number and variety of tasks posted by different requesters [27]. We advertised the survey task as “Public Opinion about Abortion Websites.” We compensated participants US $2.50 to complete the assignment. After the first 425 responses, we increased compensation to US $3.00 for the final 75 responses in order to increase recruitment. Participants were limited to responding to the survey once based on their unique worker ID number that is assigned by Amazon and is attached to their taxpayer number [27]. The study was reviewed and approved by the Oregon Health & Science University institutional review board.

### Website Selection

We selected our websites from a database of abortion websites used in a previous study [10]. For each website in that database, three investigators determined the slant (prochoice, neutral, or antichoice) and slant clarity (easy, moderate, or difficult to determine website slant) based on their impression of the website. All three researchers have published abortion-related research. We then selected 11 websites categorized as both “antichoice” and “difficult to determine” in slant (Multimedia Appendix 1). These 11 antichoice websites included one state government website (Alaska Department of Health and Social Services), four CPC pages associated with brick and mortar establishments, and six websites that detail abortion risks and side effects. We included one website that was rated as being “neutral” in slant and “easy to determine” in slant clarity (The Louisiana Department of Public Health website on “Methods & Medical Risks”). We selected the Louisiana website as a comparator for the antichoice websites.

We used previously validated studies measuring website trust [17-20,24,25] to develop our survey (Table 1). We included items across the following four domains: creators, design, content, and overall trust (Table 1). Participants viewed each website and responded to statements about website characteristics using a 7-point Likert scale ranging from −3 (“strongly disagree”) to 3 (“strongly agree”) [20,21,25,28]. We assigned each participant to assess three randomly selected antichoice websites and the Louisiana website. Therefore, all participants viewed the Louisiana website but only a subset of participants viewed each of the 11 antichoice websites. Our survey displayed all four websites in random order, and users had 18 minutes to complete the survey and rate all four websites.

**Table 1:** Website questions.

Category	Question number	Question tag	Statement about the website characteristic
Creators	q0	Creator confidence	I believe that the creators of this website are honest and trustworthy.
Creators	q1	Expert quote	There are experts quoted or referenced on this website.
Creators	q2	Organization	This website notes an association with a trustworthy organization.
Creators	q3	Expert info	Experts wrote information on this website.
Creators	q4	Nonprofit	This website is run by a nonprofit organization.
Design	q5	Interactive	I like the interactive aspects of this website.
Design	q6	Images	I like the use of images on this website.
Design	q7	Up to date	This website appears up to date.
Design	q8	Ads	I can distinguish ads from website content.
Design	q9	Look	I like the look and feel of this website.
Design	q10	Features	This website includes all relevant features/functionalities that I value on websites.
Design	q11	Design	The design of this website is similar to websites I like to use.
Design	q12	Professional	This website appears professional.
Design	q13	Navigate	This website is easy to navigate.
Content	q14	Errors	This website contains errors (spelling or grammar, not accuracy of content).
Content	q15	Address	This website provides an address or phone number.
Content	q16	Contact	This website makes it easy to contact its creators.
Content	q17	Promotional	This website has limited advertising or other promotional material.
Overall trust	q18	Info trust	I trust information from this website.
Overall trust	q19	Quality	Overall, this website is a quality resource.
Overall trust	q20^a^	Biased	This website is biased.
Overall trust	q21	Recommend	I would recommend this page to a friend who is searching for information on this topic.

^a^Question 20 was removed from the overall trust domain owing to poor correlation with other overall trust questions.

We compiled our primary outcome (“mean overall trust”) by averaging three of the four items comprising the overall trust domain. During analysis, we excluded one item “this website is biased” from the primary outcome owing to low correlation with the other questions in the trust domain. We reverse coded responses to items 14 “this website contains errors” and 20 “this website is biased,” so that all positive Likert scale responses become markers of a favorable characteristic.

After participants evaluated all four websites (responses were not required to move on to the next question), we asked sociodemographic questions, including questions on age, race/ethnicity, gender, educational background, state of residence, residential density (rural, urban, and suburban), and internet use (hours per week online) [29]. Unfortunately, an error in the MTurk survey assigned Alabama to those who did not respond to the item regarding state of residence. Finally, we asked about views on abortion, including whether abortion should be legal (no law limiting abortion, some limits, severe limits, or should be illegal in all circumstances) and self-rated abortion knowledge (1-10, with 1 being no knowledge and 10 being expert knowledge).

### Analysis

We used RStudio Version 1.2.1335 for all analyses [30]. We used descriptive statistics to characterize the overall demographics of our participants. Due to small numbers, we collapsed race and ethnicity into a binary category (white vs not white). We then conducted analyses for individual items and domains at the website level and multivariate analysis to determine relationships between demographic variables and overall trust.

For each website, we calculated mean scores for responses to the individual items as well as composite means for the four domains, including the primary outcome of mean overall trust. We tested bivariate associations between mean overall trust and each survey item from Table 1. We used correlation coefficients to measure the strength of the relationship of each individual item in the survey with the items comprising the “overall trust” category (info trust, quality, and recommend) as well as mean overall trust itself. Correlation coefficient scores range from “1” (perfect positive correlation) to “−1” (perfect negative correlation), with “0” meaning there is no correlation between two items. In order to test the robustness of our findings, we assessed these correlations in the following three ways: for all 12 websites combined, for the antichoice websites combined, and for the prochoice Louisiana website alone. We set 0.5 as the cutoff for a “marked” correlation and considered coefficients between 0.4 and 0.5 as indicating “medium” correlation [31].

Last, we used linear regression to identify participant characteristics associated with mean overall trust, where mean overall trust was treated as a continuous variable. We excluded participants with missing items in the trust domain from multivariate modeling (36/498). We created two models. One model was created for the antichoice websites, and another for the Louisiana website alone in order to compare model findings stratified by slant. Variables in our model included race, age, self-rated abortion knowledge, abortion views, hours spent on the internet per week, educational attainment, and residential density. We selected these covariates based on bivariate relationships and prior literature suggesting that these variables impact website trust [24].

## Results

### Sample

We obtained surveys from 500 participants and excluded only two respondents who did not complete 50% of the website survey questions. We included all other partial responses. Of the 498 respondents, 403 (80.9%) completed all questions for all four websites. Of the 95 incomplete surveys, 53 (56%) missed only one item on one website and fully completed the other three website evaluations. An additional 20 (21%) missed two or three items total across the four websites. A total of 6 (6%) participants missed all 22 items from one website but completed all items from the other three websites.

### Participant Characteristics

Full demographic characteristics are shown in Table 2. Respondent age ranged from 22 to 70 years, and 50.1% (247/493) identified as female. Just over half (279/495, 56.3%) of the respondents attained at least a bachelor’s degree. The majority of our respondents were white (327/495, 66.1%), and an additional 20% identified as Asian. Participants were distributed across the US census region, with 16.7% (83/498) from the Northeast, 34.7% (173/498) from the South, 19.5% (97/498) from the Midwest, 20.1% (100/498) from the West, and 9.1% (45/498) not from the United States (Table 2). The MTurk error makes it likely that 34.7% is higher than the true proportion of respondents who are from the South. On average, respondents reported spending 35.6 hours per week on the internet (95% CI 1.0-72.8). The majority of participants self-identified as prochoice, with 80.3% (399/497) indicating that there should only be some limits or no law limiting access to abortion. We found self-reported abortion knowledge in our participants to be normally distributed with a mean of 5.9/10 (95% CI 2.4-9.4).

**Table 2:** Sample characteristics.

Demographics		Value, n (%) or mean (SD; min-max)
**Gender (N=493)**		
	Female	247 (50.1)
	Male	245 (49.7)
	Other	1 (0.2)
**Education (N=495)**		
	Less than high school and high school	60 (12.1)
	Some college/associates	156 (35.12)
	Bachelor’s degree	213 (43.0)
	More than bachelor’s degree	66 (13.3)
**Location (N=489)**		
	Rural	100 (20.5)
	Suburban	215 (44.0)
	Urban	174 (35.6)
**Region (N=498)**		
	Northeast	83 (16.7)
	South^a^	173 (34.7)
	Midwest	97 (19.5)
	West	100 (20.1)
	Not United States	45 (9.1)
**Race (N=495)**		
	White	327 (66.1)
	Non-white	168 (33.9)
**Access (N=497)**		
	No law	182 (36.6)
	Some limits	217 (43.7)
	Severe limits	68 (13.7)
	Illegal	30 (6.0)
**Other**		
	Age (years)	37.9 (9.9; 22-70)
	Abortion knowledge score	5.9 (1.8; 1-10)
	Internet hours	35.6 (19.0, 1-107)

^a^Those who left the region question blank were coded as from Alabama and subsequently the South.

### Website Outcomes

Mean scores were calculated for each of the four domains (content, creators, design, and overall trust). The mean score of the overall trust domain is the primary outcome of mean overall trust. Domain scores ranged from −0.66 to 1.89, where −3 is highly unfavorable and 3 is highly favorable (Table 3). For the primary outcome of mean overall trust, the Louisiana State Health website had the highest score (1.89) and smallest standard deviation (1.27) (Table 3). In addition, the Louisiana State Health website had the lowest standard deviations in the content (1.27) and design (1.65) domains. By comparison, the Alaska State Health website received the highest score among all websites for creators (1.41), a high score for mean overall trust (1.63), and lower scores for content (1.05) and design (0.97) (Table 3).

**Table 3:** Responses by domain.

Website	Domain score^a^, mean (SD)			
	Content	Creators	Design	Mean overall trust
Louisiana^b^	1.77 (1.44)	1.31 (1.64)	1.30 (1.65)	1.89 (1.27)
Abortion Facts	0.23 (1.80)	0.32 (1.88)	0.11 (1.88)	−0.24 (1.79)
Abortion Pill Risks	0.30 (1.70)	0.39 (1.91)	0.48 (1.91)	−0.04 (1.85)
Abortion Risks	0.41 (1.71)	0.99 (1.71)	0.32 (1.91)	0.49 (1.79)
Alaska	1.05 (1.70)	1.41 (1.58)	0.97 (1.77)	1.63 (1.38)
AmerPreg	1.26 (1.54)	0.97 (1.76)	1.04 (1.67)	0.92 (1.72)
Baby Gaga	0.02 (1.71)	−0.15 (1.64)	0.56 (1.80)	0.09 (1.80)
CareNet	1.61 (1.43)	0.55 (1.67)	1.16 (1.64)	0.92 (1.61)
PregCenter	1.77 (1.42)	0.82 (1.72)	1.61 (1.31)	1.45 (1.49)
RamaInternat	0.59 (1.75)	0.37 (1.80)	0.67 (1.80)	0.37 (1.89)
U Pregnancy	−0.14 (1.67)	−0.54 (1.62)	−0.23 (1.83)	−0.66 (1.59)
WomenRes	1.70 (1.46)	0.70 (1.89)	1.05 (1.71)	1.11 (1.73)

^a^For each item, the maximum rating is +3 and minimum rating is −3.

^b^The Louisiana website is displayed as a neutral website.

Eight of the 11 antichoice websites were associated with a positive mean overall trust score (Table 3). Abortion Facts, Abortion Pill Risks, and U Pregnancy received negative scores for mean overall trust, ranging from −0.66 to −0.04. In addition, Baby Gaga received a score of −0.15 in the creators domain and U Pregnancy received scores of −0.14 for the content domain, −0.54 for the creators domain, and −0.23 for the design domain.

Pooled correlation coefficient matrices were used to compare each item to the three items included in the primary outcome of mean overall trust as well as to the primary outcome of mean overall trust (Table 4, Table 5, and Table 6). We assessed if the correlation between website characteristics and mean overall trust was different for the antichoice websites and the neutral Louisiana website. For both the antichoice websites and the Louisiana website, the creator confidence item (“I believe that the creators of this website are honest and trustworthy”) correlated most highly with mean overall trust (Table 5 and Table 6). For the Louisiana website, we found that the creator confidence item was the only item markedly correlated with mean overall trust (coefficient=0.66) (Table 6). In contrast, for the antichoice websites, professional appearance (coefficient=0.59), look and feel (coefficient=0.59), perception that the information is created by experts (coefficient=0.59), association with a trustworthy organization (coefficient=0.58), valued features and functionalities (coefficient=0.54), and interactive capabilities (coefficient=0.52) all demonstrated marked relationships with mean overall trust. Up-to-date appearance (coefficient=0.50), overall design (coefficient=0.49), ability to navigate the website (coefficient=0.48), and presence of images (coefficient=0.46) had medium correlation with mean overall trust. We found no correlation between presence of spelling or grammar errors and mean overall trust (coefficient=−0.07) (Table 5).

**Table 4:** Correlation coefficients between items in the primary outcome of mean overall trust and all individual items for all websites.

All individual items	Items in the primary outcome			
	Info trust	Quality	Recommend	Mean overall trust
Info trust	1.00	0.86	0.79	0.88
Quality	0.86	1.00	0.79	0.88
Recommend	0.79	0.79	1.00	0.86
Creator confidence	0.76	0.74	0.65	0.72
Professional	0.61	0.64	0.59	0.61
Expert info	0.63	0.61	0.58	0.61
Organization	0.60	0.61	0.57	0.60
Look	0.57	0.59	0.58	0.58
Features	0.53	0.56	0.53	0.54
Up to date	0.52	0.55	0.50	0.52
Navigate	0.49	0.52	0.46	0.49
Design	0.47	0.48	0.48	0.48
Interactive	0.47	0.47	0.48	0.47
Address	0.43	0.43	0.44	0.43
Contact	0.40	0.43	0.42	0.42
Promotional	0.43	0.42	0.38	0.41
Images	0.37	0.38	0.38	0.38
Biased	0.39	0.37	0.29	0.35
Expert quote	0.34	0.34	0.31	0.33
Nonprofit	0.34	0.31	0.29	0.31
Ads	0.27	0.29	0.27	0.28
Errors	−0.06	−0.05	−0.07	−0.06

**Table 5:** Correlation coefficients between items in the primary outcome of mean overall trust and all individual items for all websites except Louisiana.

All individual items	Items in the primary outcome			
	Info trust	Quality	Recommend	Mean overall trust
Info trust	1.00	0.85	0.78	0.88
Quality	0.85	1.00	0.77	0.87
Recommend	0.78	0.77	1.00	0.85
Creator confidence	0.74	0.73	0.64	0.70
Professional	0.58	0.62	0.58	0.59
Look	0.58	0.60	0.58	0.59
Expert info	0.62	0.59	0.56	0.59
Organization	0.59	0.60	0.55	0.58
Features	0.53	0.57	0.53	0.54
Interactive	0.51	0.53	0.52	0.52
Up to date	0.49	0.52	0.48	0.50
Design	0.48	0.50	0.49	0.49
Navigate	0.48	0.51	0.44	0.48
Images	0.46	0.46	0.46	0.46
Contact	0.37	0.40	0.38	0.38
Address	0.37	0.36	0.39	0.37
Promotional	0.39	0.38	0.35	0.37
Expert quote	0.37	0.37	0.34	0.36
Biased	0.38	0.36	0.28	0.34
Nonprofit	0.37	0.33	0.31	0.34
Ads	0.26	0.29	0.27	0.27
Errors	−0.07	−0.06	−0.08	−0.07

**Table 6:** Correlation coefficients between items in the primary outcome of mean overall trust and all individual items for Louisiana alone.

All individual items	Items in the primary outcome			
	Info trust	Quality	Recommend	Mean overall trust
Quality	0.82	1.00	0.70	0.84
Info trust	1.00	0.82	0.69	0.84
Recommend	0.69	0.70	1.00	0.80
Creator confidence	0.72	0.69	0.56	0.66
Expert info	0.49	0.48	0.42	0.46
Professional	0.45	0.51	0.41	0.46
Up to date	0.46	0.47	0.41	0.45
Organization	0.44	0.44	0.42	0.43
Features	0.43	0.43	0.43	0.43
Look	0.39	0.38	0.43	0.40
Navigate	0.41	0.42	0.37	0.40
Design	0.31	0.32	0.34	0.32
Promotional	0.31	0.35	0.25	0.30
Interactive	0.28	0.26	0.33	0.29
Expert quote	0.28	0.26	0.23	0.26
Address	0.22	0.27	0.29	0.26
Contact	0.18	0.25	0.28	0.24
Ads	0.20	0.25	0.19	0.21
Images	0.14	0.16	0.17	0.16
Nonprofit	0.16	0.14	0.15	0.15
Biased	0.20	0.17	0.09	0.15
Errors	0.00	0.01	−0.05	−0.01

### Multivariate Analysis

We performed multivariate analysis to identify associations between participant demographics and the primary outcome of mean overall trust. We found that preference for fewer abortion restrictions was associated with higher mean overall trust of the Louisiana website (*B*=−0.43, 95% CI −0.87 to 0.01) and with lower mean overall trust of the antichoice websites (*B*=0.52, 95% CI 0.05-0.99) (Table 7 and Table 8). In addition, white race was associated with lower mean overall trust of the antichoice websites (*B*=−1.41, 95% CI −2.26 to −0.55) (Table 8). Other participant characteristics were not associated with mean overall trust.

**Table 7:** Association between individual demographic factors and the primary outcome of mean overall trust (N=462) for the Louisiana website.

Variable	Estimate	95% CI
Intercept	5.16	2.75 to 7.57
White race	0.36	−0.45 to 1.16
Age	0.004	−0.03 to 0.04
Abortion knowledge	0.14	−0.05 to 0.32
Internet hours	0.0003	−0.02 to 0.02
Abortion access	−0.43	−0.87 to 0.01
Education	0.09	−0.32 to 0.50
Urban residence	0.05	−0.90 to 1.00
Suburban residence	−0.43	−1.35 to 0.49

**Table 8:** Association between individual demographic factors and the primary outcome of mean overall trust (N=462) for all websites except the Louisiana website.

Variable	Estimate	95% CI
Intercept	−0.49	−3.09 to 2.11
White race	−1.41	−2.26 to −0.55
Age	−0.008	−0.05 to 0.03
Abortion knowledge	0.33	0.13 to 0.53
Internet hours	0.01	−0.01 to 0.03
Abortion access	0.52	0.05 to 0.99
Education	0.08	−0.35 to 0.52
Urban residence	0.10	−0.92 to 1.13
Suburban residence	−0.58	−1.56 to 0.40

## Discussion

In this study, we analyzed which characteristics of antichoice websites are highly correlated with mean overall trust. User opinion of website creators had the strongest correlation with mean overall trust, but esthetic and website interface factors were also important. Moreover, even with our predominantly prochoice respondent pool, eight out of the 11 antichoice websites received positive mean overall trust scores, consistent with their selection as websites with “difficult to determine” slant. We also found that a viewer’s stance on abortion is inversely related to the mean overall trust of antichoice websites. If participants believed that abortion should be limited or banned, they were more likely to have higher mean overall trust in websites with incorrect information.

To examine the importance of website creators, we compared the high-quality neutral-stance Louisiana state health website with the poor-quality antichoice Alaska state health website. While the antichoice Alaska website received lower scores in the content, design, and overall trust domains than the neutral Louisiana website, the Alaska website received a higher score in the creators domain (1.41 vs 1.31) and was rated as the website with the second highest mean overall trust. Furthermore, we found that design items were less correlated with mean overall trust for the Alaska website than they were for the other antichoice websites. It is reasonable that users would be inclined to assume that an official state health website is trustworthy. Our results suggest that the belief that a website creator is trustworthy can override other aspects of the website with decreased vigilance to lower quality content.

To attract users, antichoice websites use a full array of internet tools that draw from all trust domains to give the impression of credibility [11]. In our study, nine out of 11 antichoice websites received a positive score for the creators domain. These websites use strategies similar to those that brick and mortar CPCs use, such as deceptive advertising, to attract patients [32]. While brick and mortar CPCs often present as though they offer services similar to abortion clinics (ultrasound, “options” counseling, and procedures), the online websites rely on website cues that resemble high-quality resource websites. For instance, many antichoice websites use generic URL titles like “americanpregnancy.org” and include features like “FAQs” that resemble accurate informational websites, but contain misleading information that misrepresents rare or impossible adverse outcomes and fail to refer patients to clinics that provide abortion care. Our findings support that these methods are effective by demonstrating that a website is more likely to have high mean overall trust scores if its appearance and design are highly rated.

Our trends are consistent with prior literature including research examining user trust in other online health contexts [17-20,24,25]. Shanley et al conducted a qualitative study on the perceptions of abortion websites and found that creator expertise and affiliation with an organization were important criteria when assessing website trustworthiness [25]. In addition, several studies describing the impact of health website characteristics on credibility noted that creator “authority” and esthetic attributes, such as design, are associated with trustworthiness [19,21,24]. Of note, several studies indicated that page ranking of search results affects user trust scores [15,20]. On Google web search, many CPC websites pay to become promoted search items and appear at the top of the page [33]. In response to CPC website tactics, reputable organizations should tackle search engine optimization, clearly identify website creators, and prioritize website design.

Our findings should be interpreted with several limitations. We only reviewed a limited sample of antichoice websites and compared them to a single standard “neutral website” in order to learn how these websites are perceived. However, our websites were selected from those most commonly found on a web search, and thus, they are more likely to be seen by the general public. Moreover, compared to prior studies that used qualitative interviews and contained small convenience samples, each one of our websites received over 130 unique evaluations from across the entire country [25]. Our participant pool leaned toward white, higher-educated, and prochoice individuals, consistent with previous studies on how MTurk workers may be demographically skewed [34-36]. However, there is evidence to suggest that MTurk studies are as reliable as studies involving other survey platforms [37,38]. Our study population was diverse as it included a nearly equal number of male and female participants, a wide age range, and participants from all US census regions. Furthermore our multivariate analysis allowed us to control for many demographic characteristics. Finally, our study did not allow us to determine if causality exists between different website factors and mean overall trust scores. For example, study participants may have chosen to give high scores to all items for websites they regarded as trustworthy. However, many of our websites had low mean overall trust scores but still received high ratings for items in other domains.

This study highlights the characteristics of antichoice websites that are most associated with user trust and demonstrates that many antichoice websites are viewed as trustworthy by a lay audience. People who seek online medical information about abortion may be susceptible to deceptive websites and misinformation. For organizations and individuals seeking to disseminate accurate information about abortion, this study underscores that attention should be directed toward highlighting the credentials of website creators in addition to providing evidence supporting website content.

## Appendix

Multimedia Appendix 1 All websites and URLs.

## Abbreviations

CPC: crisis pregnancy center
MTurk: Mechanical Turk
