# Supplementary material for: Factors Associated With Perceived Trust of False Abortion Websites: Cross-sectional Online Survey
Source: J Med Internet Res. 2021 Apr 19;23(4):e25323. doi: 10.2196/25323 (PMC8094019; doi:10.2196/25323)
Supplement: Multimedia Appendix 1 [file jmir_v23i4e25323_app1.docx]

**Multimedia Appendix 1.** All websites and URLs.

| **Website Code** | **Website Address** |
| --- | --- |
| Louisiana | http://ldh.la.gov/index.cfm/page/1036 |
| RamaInternat | <https://ramahinternational.org/abortion-risks-dangers/> |
| Alaska | <http://dhss.alaska.gov/dph/wcfh/Pages/informedconsent/abortion/risks.aspx> |
| PregCenter | <https://www.cspregnancycenter.com/side-effects-of-abortion.htm> |
| UPreg | <https://upregnancy.com/infertility-after-abortion-1443> |
| WomenRes | <https://www.womensresourceclinic.org/post-abortion-syndrome-and-symptoms/> |
| AbortionFacts | <https://www.abortionfacts.com/reardon/list-of-major-psychological-sequelae-of-abortion> |
| AmerPreg | <http://americanpregnancy.org/unplanned-pregnancy/abortion-emotional-effects/> |
| CareNet | <https://www.carenetnorcal.org/> |
| AbortionRisks | <http://abortionrisks.org/index.php?title=Depression> |
| BabyGaga | <https://www.babygaga.com/15-abortion-side-effects-and-complications/> |
| AbortionPillRisks | <http://abortionpillrisks.org/> |
